# The grading detection model for fingered citron slices (citrus medica ‘fingered’) based on YOLOv8-FCS

**DOI:** 10.3389/fpls.2024.1411178

**Published:** 2024-06-05

**Authors:** Lingtao Zhang, Pu Luo, Shaoyun Ding, Tingxuan Li, Kebei Qin, Jiong Mu

**Affiliations:** ^1^ College of Information Engineering, Sichuan Agricultural University, Ya’an, China; ^2^ Ya’an Digital Agriculture Engineering Technology Research Center, Sichuan Agricultural University, Ya’an, China

**Keywords:** finger citron, YOLOv8, object detection, smart agriculture, grading

## Abstract

**Introduction:**

Fingered citron slices possess significant nutritional value and economic advantages as herbal products that are experiencing increasing demand. The grading of fingered citron slices plays a crucial role in the marketing strategy to maximize profits. However, due to the limited adoption of standardization practices and the decentralized structure of producers and distributors, the grading process of fingered citron slices requires substantial manpower and lead to a reduction in profitability. In order to provide authoritative, rapid and accurate grading standards for the market of fingered citron slices, this paper proposes a grading detection model for fingered citron slices based on improved YOLOv8n.

**Methods:**

Firstly, we obtained the raw materials of fingered citron slices from a dealer of Sichuan fingered citron origin in Shimian County, Ya'an City, Sichuan Province, China. Subsequently, high-resolution fingered citron slices images were taken using an experimental bench, and the dataset for grading detection of fingered citron slices was formed after manual screening and labelling. Based on this dataset, we chose YOLOv8n as the base model, and then replaced the YOLOv8n backbone structure with the Fasternet main module to improve the computational efficiency in the feature extraction process. Then we redesigned the PAN-FPN structure used in the original model with BiFPN structure to make full use of the high-resolution features to extend the sensory field of the model while balancing the computation amount and model volume, and finally we get the improved target detection algorithm YOLOv8-FCS.

**Results:**

The findings from the experiments indicated that this approach surpassed the conventional RT-DETR, Faster R-CNN, SSD300 and YOLOv8n models in most evaluation indicators. The experimental results show that the grading accuracy of the YOLOv8-FCS model reaches 98.1%, and the model size is only 6.4 M, and the FPS is 130.3.

**Discussion:**

The results suggest that our model offers both rapid and precise grading for fingered citron slices, holding significant practical value for promoting the advancement of automated grading systems tailored to fingered citron slices.

## 1 Introduction

Fingered citron (citrusmedical. Var. Sarco-dactylisSwingle), a plant of the genus Citron in the family Rutaceae, is named because its fruit petals are shaped like fingers, resembling the hand of the Buddha, and is also known as Bergamot, bergamot citron, miro, and longevity oranges, which is one of the major traditional Chinese medicinal herbs in China (Wang et al., 2020). *The Pharmacopoeia of the People’s Republic of China* has included fingered citron since the 1963 edition, and it has been included until now (Zhang et al., 2018). Fingered citron has a long history of medicinal use. The fruit is used as medicine, which has the effect of regulating the flow of Qi and stopping vomiting, harmonizing the stomach and spleen, eliminating food and resolving phlegm (Luo et al., 2020). Modern medicine is even more research has proved that fingered citron has antidepressant, antibacterial, anti-inflammatory, anticancer, antitumor, antiaging, blood pressure lowering and other effects (Peng et al., 2009; Karp and Hu, 2018; Li et al., 2018). Fingered citron slices are harvested in its autumn when the fruits have not yet turned yellow or yellow, and are often primed by slicing and then sun-drying or low-temperature drying to become fingered citron slices (Klein, 2014; Wu, 2015; Chen et al., 2020).

In today’s market, fingered citron slices, as the most widely distributed fingered citron agricultural by-products, are preferred to those with large slices, green skin and white flesh, and strong aroma (Xu et al., 2021a). They are classified into three grades, namely first-grade, second-grade and third-grade. At the same time, due to the low prevalence of standardization and the fragmentation of farmers and dealers, unusable bad slices and counterfeit slices are often mixed in (Xu et al., 2021b), resulting in the loss of interests of many parties. Moreover, most of the agricultural products such as fingered citron slices are judged by laborers using manual visual estimation to determine the grade, which is inconsistent and labor-intensive (Meena et al., 2011; Ismail and Malik, 2022).The continuous loss of agricultural labor in recent years and the unprecedented increase in labor costs (Patel and Patil, 2024), which became more prominent after the COVID-19 pandemic (Nawaz et al., 2021), are detrimental to the market for fingered citron slices and the expansion of fingered citron cultivation scale. In summary, fingered citron slices grading technology, as a key factor to improve the quality of fingered citron slices and to liberate the orchard labor force, is of great significance to increase the added value of the product, to improve the competitiveness of the market, and to alleviate the shortage of orchard labor. A high-precision, high-speed and damage-free grading method is needed to grade fingered citron slices effectively and objectively.

With the continuous development of computer science and technology, computer vision technology in deep learning has penetrated into people’s production and life. More and more research has applied computer vision technology to agricultural production and has a wide range of practical applications (Chen et al., 2024a). In research focusing on the grading of agricultural goods using traditional machine learning, Castro and colleagues (Castro et al., 2019) assessed the efficacy of four distinct machine learning strategies paired with three different color spaces for the categorization of cape gooseberry fruit based on their stage of ripeness. Their findings revealed that the utilization of the Lab* color space in conjunction with a Support Vector Machine (SVM) classifier yielded the highest levels of precision and f-measure. In another study, Moallem and associates (Moallem et al., 2017) introduced an algorithm that employs computer vision to evaluate apples. This method involved extracting both textural and geometric attributes from defective regions of the apples. The fruits were then categorized into first-grade, second-grade, and irregular categories using a combination of SVM, Multi-Layer Perceptron (MLP), and K-Nearest Neighbors (KNN) classifiers. Gui et al (Gui et al., 2014). suggested a method for classifying apple shapes based on wavelet rectangles, which successfully sorted apples into three categories: normal, slightly misshapen, and severely misshapen, with respective classification accuracies of 86.2%, 85.8%, and 90.8%. Among these machine learning-based grading techniques, preprocessing of images is a common necessity, and the reliance on singular features for classification can lead to challenges such as suboptimal real-time performance and diminished robustness.

In a research project centered on fruit grading using deep learning techniques, Chakraborty and colleagues (Chakraborty et al., 2023) crafted a specialized, lightweight Convolutional Neural Network (CNN) known as “SortNet”. This model is designed for straightforward implementation on edge devices to facilitate real-time citrus fruit sorting through visual analysis. The model’s performance, as demonstrated on a test dataset, is detailed in the accompanying table, highlighting an impressive accuracy rate of 97.6%. Chen et al (Chen et al., 2024b. introduced a multi-task Deep Convolutional Neural Network (DCNN) detection model, MTD-YOLOv7, which is an enhancement of the YOLOv7 framework. This model is tailored for ripeness detection in cherry tomato clusters and has achieved a composite score of 86.6% in multi-task learning, with an average inference time of merely 4.9 milliseconds. Momeny and team (Momeny et al., 2020) enhanced the CNN’s generalization capabilities by integrating Max-pooling and Average-pooling techniques for cherry classification. The CNN model delivered classification accuracy of 99.4%, 98.7%, and 99.1% for various input image dimensions. Fan et al (Fan et al., 2020). employed a Convolutional Neural Network (CNN) to recognize apple quality. After training the CNN, they achieved a 96.5% accuracy on the test set. They developed a CNN-based classification software and integrated a computer vision module into a four-threaded fruit sorting machine, capable of sorting at a pace of five fruits per second, with an overall classification accuracy of 92%. However, the model’s size is considerable, leading to relatively low computational efficiency. Raikar et al (Raikar et al., 2020). explored the quality classification of okra, categorizing it into four size-based types: small, medium, large, and extra-large. They utilized three deep learning models—AlexNet, GoogLeNet, and ResNet50—with the ResNet model achieving a remarkable accuracy exceeding 99%. Luna et al (De Luna et al., 2019). proposed a deep learning-based approach for identifying defective areas in individual tomatoes. This method utilized the OpenCV library and Python programming. They collected 1200 tomato images of varying qualities using an image capture box. These images were used to train VGG16, InceptionV3, and ResNet50 deep learning models. Upon comparing the experimental outcomes, they determined that VGG16 is the most effective deep learning model for defect detection. Asriny et al (Asriny et al., 2020). suggested a deep learning-based CNN model for categorizing orange images into five distinct classes. They experimented with ReLU and Tanh activation functions, finding that the ReLU activation function surpassed Tanh in the hidden layer. The classification results using the ReLU activation function for data training showed an accuracy of 98.6%, while the validation data yielded 92.8%, and the test data achieved 96%. Fu and associates (Fu et al., 2022) constructed a linear regression model to assess and measure fruit freshness by analyzing the darkness and color changes in the fruit’s skin. They evaluated a range of fruits, including apples, bananas, dragon fruits, kiwis, oranges, and pears, for freshness grading, and the highest average accuracy attained was 96.34%. The results indicated that deep learning algorithms are highly effective in addressing this issue. Gururaj and colleagues (Gururaj et al., 2023) developed an innovative CNN architecture for the in-depth grading of three mango varieties. They achieved an accuracy of 93.23% for variety identification and an impressive 95.11% for quality grading.

However, the above deep learning model grading methods still have problems such as insufficient model optimization, poor real-time performance, and are less applied to herbal medicines such as fingered citron slices. Drawing from this foundation, the present study focuses on fingered citron slices as the subject of investigation and delves into the development of an efficient grading detection system specifically tailored for these segments. The paper proposes a fingered citron slices grading detection algorithm based on an improved YOLOv8n, using FasterNet instead of the original YOLO8 backbone, which consumes fewer memory accesses and tends to have higher FLOPS, in order to achieve an improved computational efficiency of the model. The architecture of the initial model is re-envisioned by incorporating the BiFPN (Bi-directional Feature Pyramid Network) module, enhancing the model’s capacity for feature integration while simultaneously eliminating superfluous connections to achieve a more streamlined design. Experiment data indicates that this refined approach successfully elevates the detection accuracy of the model, all without incurring additional costs in the training process. Finally, we substantiated the method’s viability and dependability through a series of ablation studies and rigorous statistical analyses. The outcomes substantiate that the YOLOv8-FCS algorithm, as introduced in this work, is adept at executing swift and precise grading of fingered citron slices. It can provide a relatively authoritative grading standard and the basis of counterfeit detection for the fingered citron slices market, and help to promote the development of automation in the fingered citron industry.

## 2 Materials and methods

### 2.1 Dataset construction

**Figure 1:**
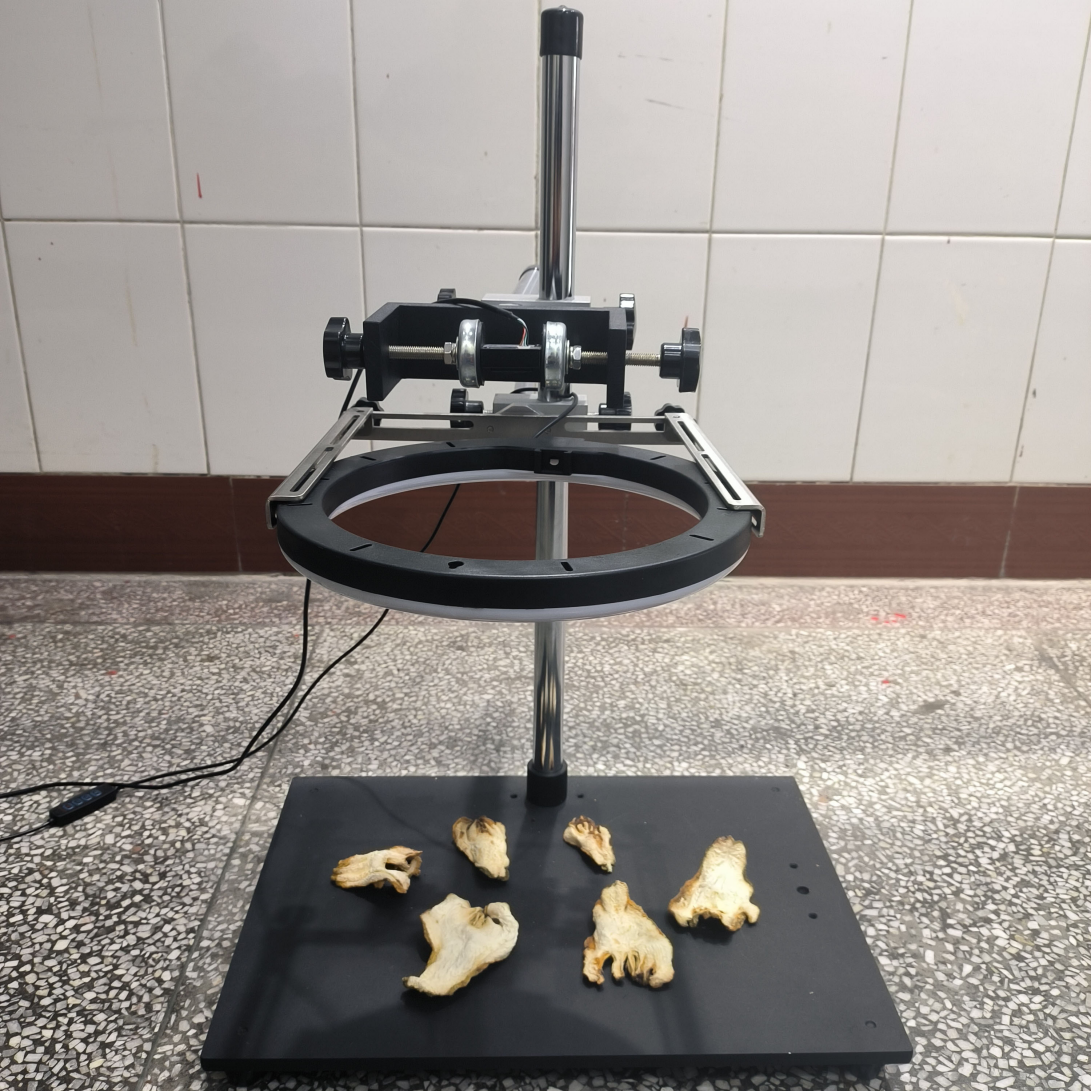
Shooting platform.

Fingered citron grows in most of the warm citrus producing areas of China (Karp and Hu, 2018). The fingered citron slices used in this dataset were obtained from a Sichuan fingered citron origin dealer in Shimian County, Ya’an City, Sichuan Province, China. The image acquisition devices used in the experiment were Canon camera (60D, 18 megapixel DIGIC4, 18–200 IS lens), iphone15, and Redmi K50 (Xiaomi Technology Co. Ltd., Beijing, China) in order to simulate a variety of filming devices in the real application environment. The above equipment is installed on a self-made shooting platform to shoot at a fixed angle, and partially uses LED light strips as supplementary light sources to capture images of fingered citron slices under diffuse illumination. The fixed position for shooting is 40 centimeters away from the sample. The shooting platform is shown in **Figure 1**.

According to the 2020 edition of *Chinese Pharmacopoeia*, *Identification of Traditional Chinese Medicines*, *Introduction to the Identification of Practical Chinese Medicinal Traits* and *Instructions for Clinical Use of the Pharmacopoeia of the People’s Republic of China*, fingered citron slices are preferable to those with large slices, green skin and white flesh, and strong aroma. It can be seen that the original medicinal materials are preferred to those with heavy quality in terms of commercial specifications. In the traditional grade evaluation, the appearance of the skin-type tablets is required to be thick skin, fine silk and excellent quality. In conclusion, fingered citron slices are preferable to the heavy ones, and the external form is mainly concerned with its quality and thickness. On the other hand, fingered citron slices is preferable to thick skin and thin silk, and its external form is mainly concerned with quality and width. Therefore, the fingered citron slices collected are classified into 4 grades with reference to relevant industry standards and experts’ experience.

**Figure 2:**
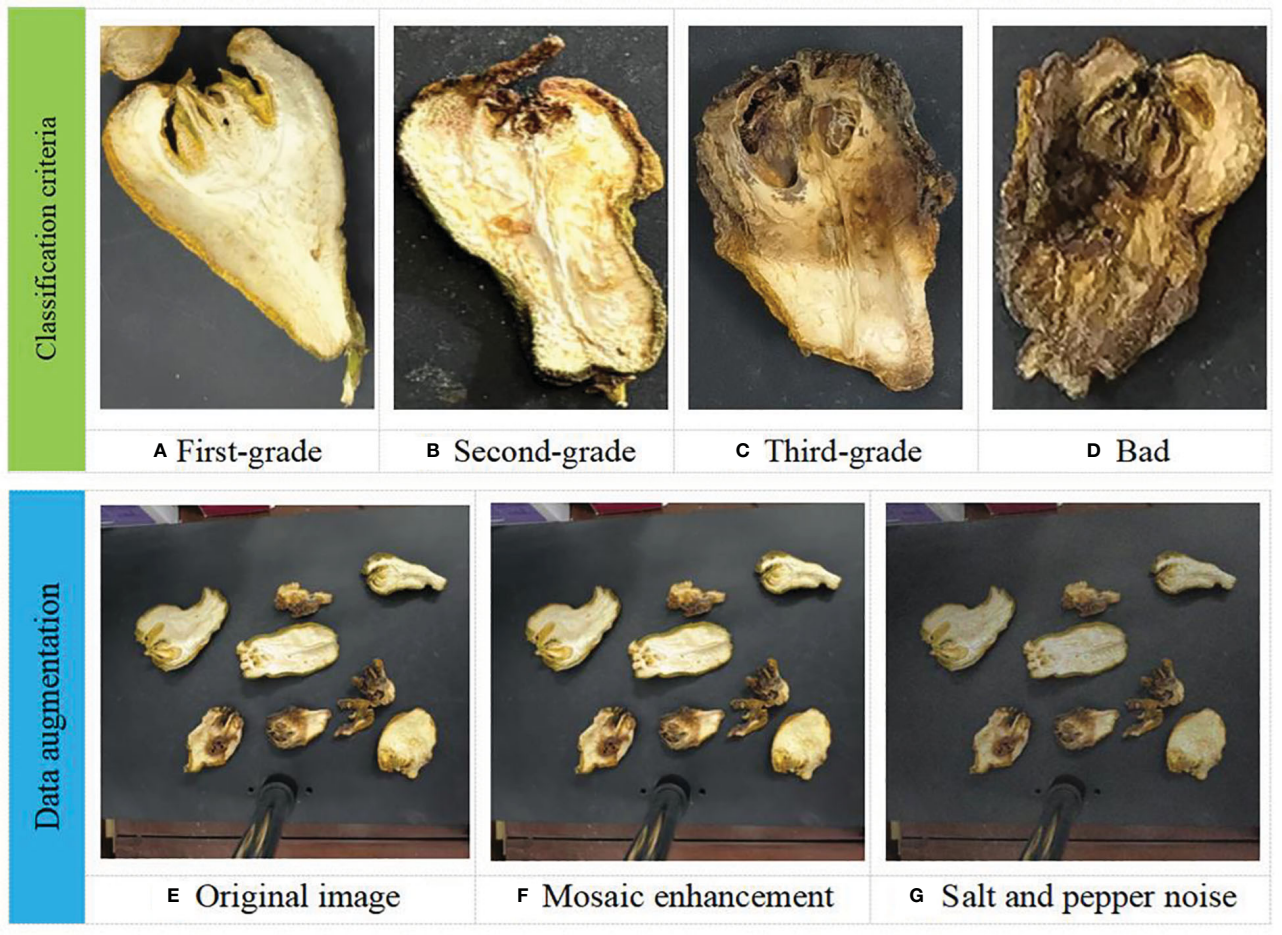
Four levels of fingered citron slices **(A–D)** and images before and after data augmentation **(E–G)**.

First-grade products are often packaged as selected pieces and enter the market, with the highest selling price. Second-grade and third-grade products are bergamot slices with some defects or long storage life, usually entering the market as a single piece, with significant price fluctuations. Bad products include moldy, blackened bergamot slices, or excessively baked scraps, which are usually not sold. Therefore, under the guidance of experts, we classify the bergamot slices based on their color and morphological characteristics. The classification criteria are as follows:

- The color of the first-grade products are slightly yellow, with a regular shape and almost no damage or defects, as shown in **Figure 2A**.
- The color of the second-grade products are slightly yellow, with a relatively regular shape and slight damage or defects, as shown in **Figure 2B**.
- The third-grade products have a brown color, irregular shape, and obvious damage or defects, as shown in **Figure 2C**.
- The color of the bad products are black brown, with an irregular shape and scattered damages or defects, as shown in **Figure 2D**.

After we manually filtered out some duplicate and similar data to simulate real application scenarios, we used Gaussian blur and salt and pepper noise to enhance our dataset to improve the generalization and robustness of the model. Examples of four levels of fingered citron and images before and after data augmentation are shown in **Figures 2E–G**.

Finally we selected 609 images as our initial dataset. We then labeled the dataset according to the above criteria using the open source script LabelImg on GitHub under the guidance of experts, and divided the dataset into training, validation, and test sets according to the ratio of 6:2:2. The labeling process produced a total of 4755 labeled frames, including 1911 first-grade labeled frames, 1620 second-grade labeled frames, 663 third-grade labeled frames, and 561 bad labeled frames. The division of the dataset and the distribution of the number of labeled frames are shown in **Table 1**.

**Table 1:** Partition of dataset and distribution of labeled boxes.

Data	Proportion	Number of images	Number of labels
Training set	60%	365	2832
Validation set	20%	122	975
Testing set	20%	122	948
sum	100%	609	4755

### 2.2 Design of fingered citron slices grading method based on improved YOLOv8n

#### 2.2.1 FasterNet

Past means of lightweighting are mainly based on Deep Separable Convolution (DWConv) (Howard et al., 2017). DWConv is a commonly used operation in convolutional neural networks that reduces the number of parameters (FLOPs) and computational complexity by dividing the convolution into two steps: deep convolution and point-by-point convolution. Deep convolution performs the convolution operation independently for each input channel, capturing features on different channels. While point-by-point convolution performs inter-channel combination and fusion of feature maps through a 1×1 convolution kernel. DWConv has the advantages of reduced number of parameters, reduced computational complexity and improved feature expression, making it an efficient convolution operation. However, the nature of its channel-by-channel convolutional operation results in the need for frequent memory accesses in practical operation. This makes the floating-point operation speed (FLOPS) of DWConv become low, which reduces the actual running speed of this operation. Chen et al (Chen et al., 2023). further proposed FasterNet in view of the new PConv and the off-the-shelf PWConv as the main operators.

We found that GhostNet has a certain degree of redundancy in the convolutional channels (Han et al., 2020). FasterNet uses standard convolution on this basis, but processes only part of the channels, and the features of the other part of the channels are directly mapped as constant. In this way, the high FLOPS advantage of standard convolution is utilized, while the FLOPs of normal convolution are reduced due to processing only part of the channels.

The main structure of FasterNet that The FasterNet Block is designed with three parts. The feature input first passes through PConv, which processes only part of the features, followed by 1×1 Conv to expand the number of channels to twice. After going through BN and ReLU, another 1×1 Conv reduces the number of channels and finally a residual link is done.

**Figure 3:**
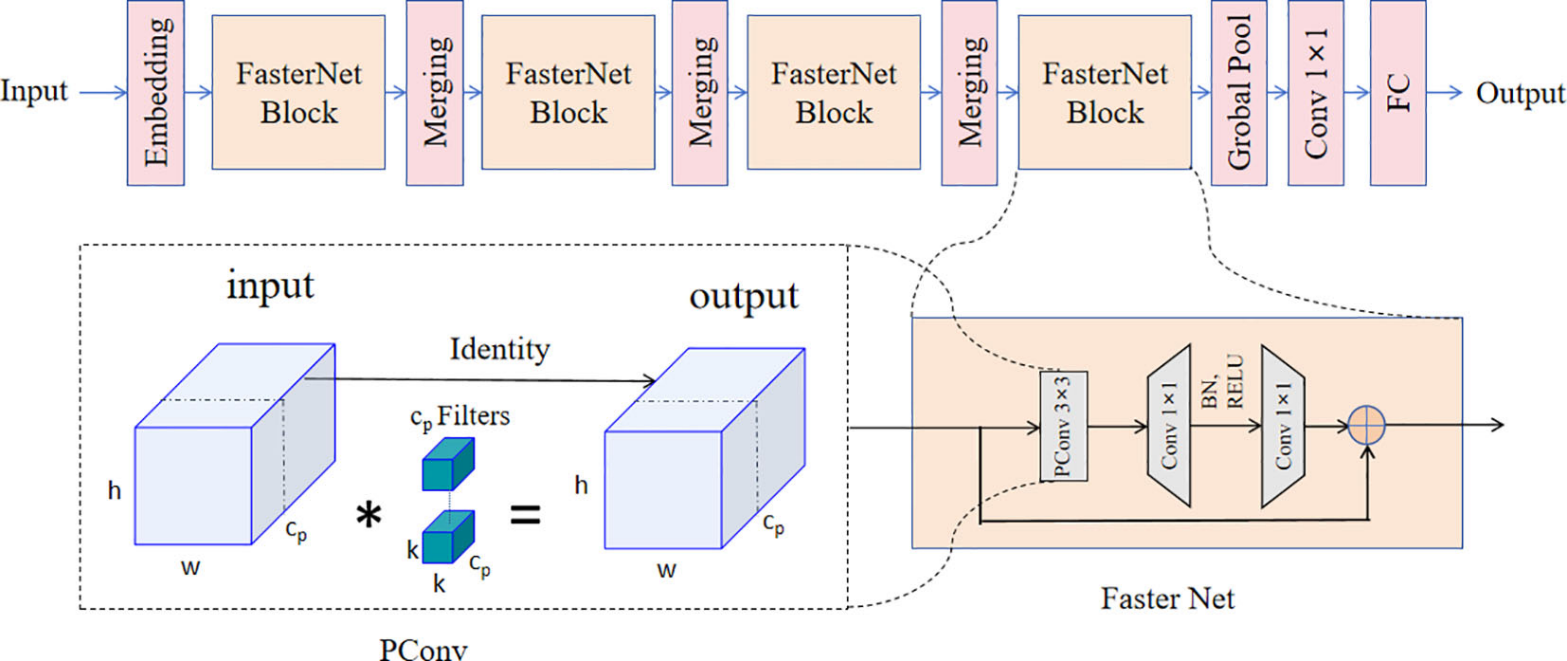
The structure of FasterNet.

The overall FasterNet architecture is shown in the **Figure 3**. It has 4 hierarchical stages, Each level is initiated by either an embedding layer, which consists of a standard 4×4 convolution with a stride of 4, or a merging layer, featuring a standard 2×2 convolution with a stride of 2, both of which are utilized to achieve spatial downsampling and an increase in the channel count. Within each hierarchical level, a multitude of FasterNet blocks is integrated. Given that the blocks in the penultimate and final stages demand less memory and are inclined to offer greater floating-point operations per second (FLOPS), there is an increased deployment of these blocks, with a corresponding augmentation in computational resources dedicated to these stages. A typical FasterNet block commences with a PConv layer, which is then succeeded by 2 PWConv layers or 1×1 convolutional layers, they form an inverted residual configuration. In this setup, the intermediate layer boasts an expanded channel capacity, and a shortcut connection is implemented to redeploy the features from the input. FasterNet constitutes a novel breed of neural networks, renowned for their rapid execution. It excels at a variety of visual tasks, benefited by its streamlined architecture that is both user-friendly and compatible with a broad spectrum of hardware.

#### 2.2.2 BiFPN

After replacing the backbone structure with the FasterNet structure, although it can significantly improve the model mAP, the inference time and model size have increased. Therefore, we expect to find a more efficient way of feature fusion as a way to achieve lightweighting and improve accuracy.

**Figure 4:**
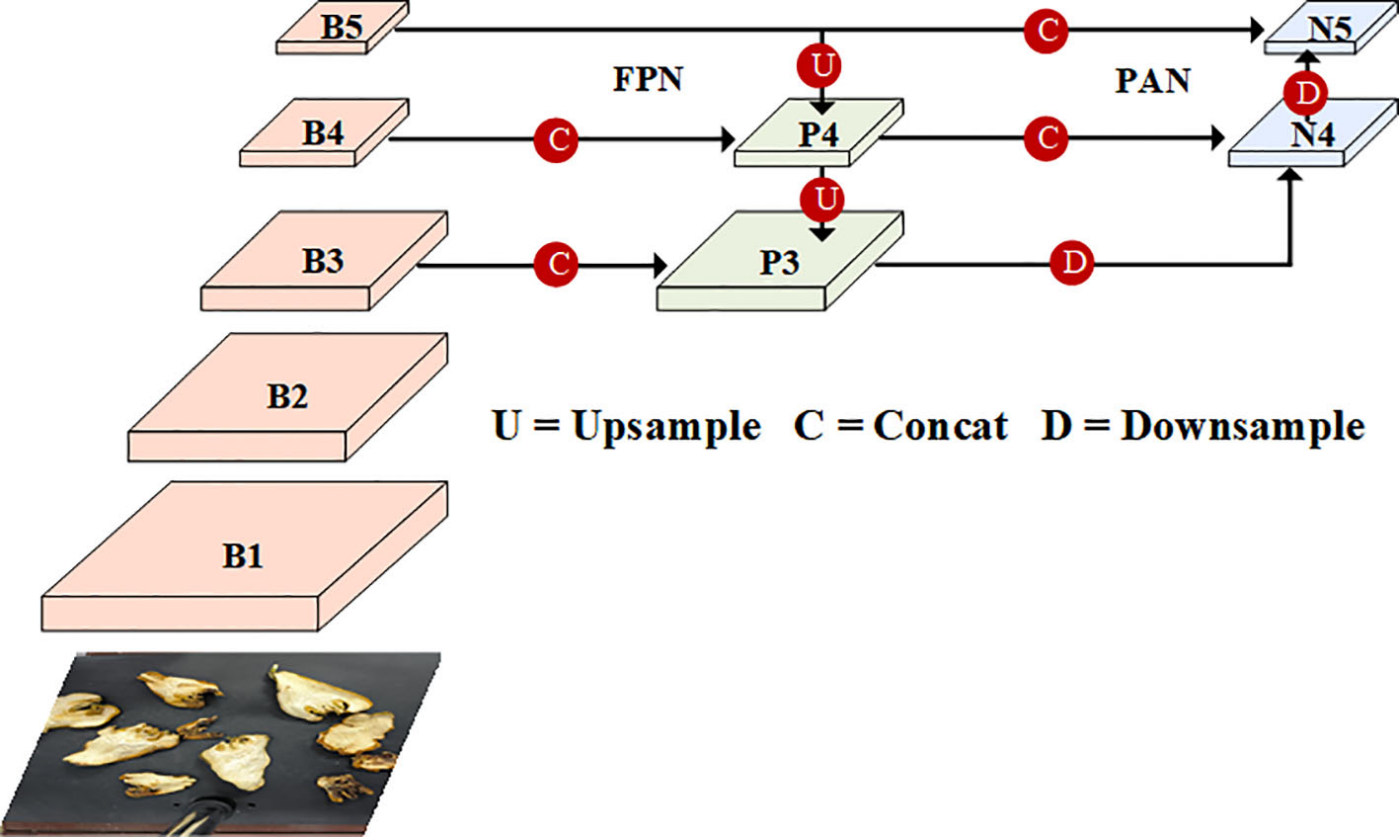
The structure of PAN-FPN.

During training, the surface defect targets of fingered citron slices have different shapes and sizes, resulting in features with different resolution sizes. Inspired by PAnet (Liu et al., 2018), the neck of YOLOv8 is designed as a PAN-FPN structure. The regular linear superposition of these features lead to an uneven weighting of features in the fusion output associated with surface defect targets in different fingered citron slices. This imbalance lead to the dominance of large-scale features in the post-fusion output, overwhelming smaller features to influence the grading judgment of fingered citron slices. The structure of PAN-FPN is shown in **Figure 4**.

To address this challenge and bolster the Neck network’s capacity to manage intricate features, it is imperative to select a proficient feature fusion network that can effectively process the features extracted by the feature extraction network across various layers.

**Figure 5:**
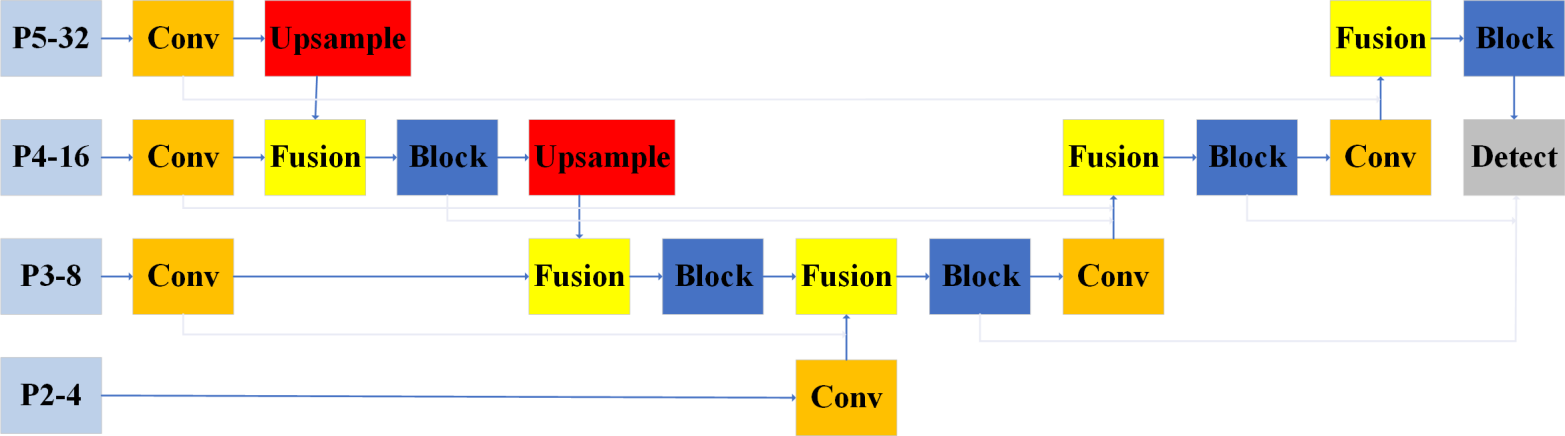
The structure of BiFPN.

The architecture of the BiFPN was initially conceived by Google as part of the EfficientDet (Tan et al., 2020) object detection algorithm. We have adopted BiFPN to serve as the neck network in our model, effectively superseding the previous PAN-FPN framework utilized in YOLOv8. **Figure 5** illustrates the configuration of the BiFPN structure.

The BiFPN framework offers an enhanced capacity for feature fusion and more efficient inter-scale connectivity when compared with the PAN-FPN structure. BiFPN streamlines the network by pruning nodes that possess a single input edge, as these have a minimal impact on the comprehensive feature network. Moreover, BiFPN refines the feature integration process by establishing additional connections between the initial input and the output nodes, and by iterating the bi-directional pathway across the same layer multiple times. This approach facilitates robust feature fusion while maintaining a relatively low increment in computational expenditure. It should be noted that the fusion module used in this experiment is a weighted fusion model. In other words, the feature maps are added directly in the spatial and channel dimensions.

The core idea of BiFPN structure is to utilize the information flow in both bottom-up and top-down directions to construct the feature pyramid. Meanwhile, a repetition-weighted fusion method is used at each pyramid level. By utilizing the information flow from both directions, the BiFPN structure can fuse features at different levels to better fit objects of various sizes. By repeating the weighted fusion process, the BiFPN structure enhances the accuracy and generalization of the model, which leads to improved target detection performance.

#### 2.2.3 YOLOv8-FCS deep learning network structure

**Figure 6:**
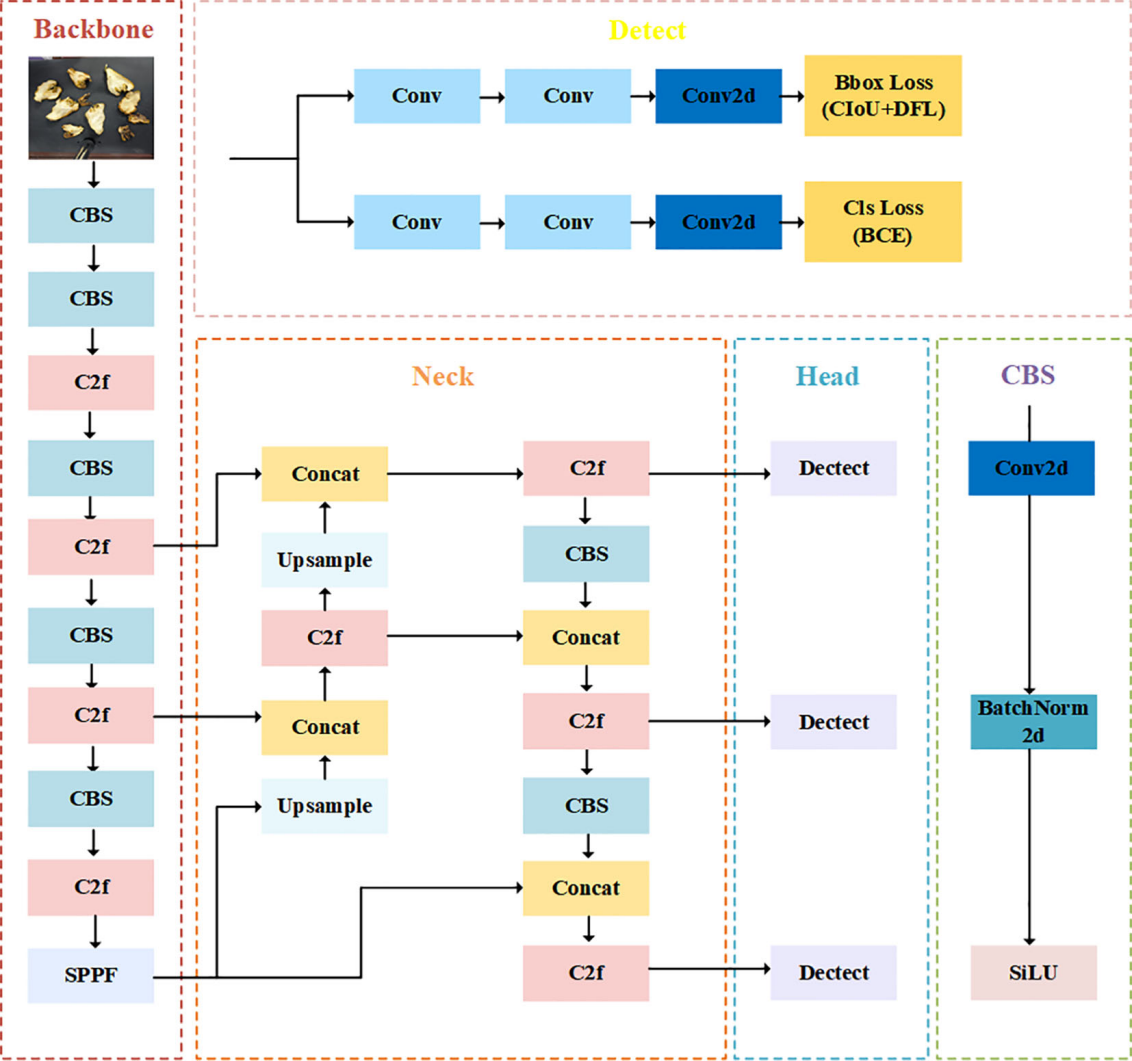
The main structure of YOLOv8.

YOLO (You Only Look Once) is a typical single-stage target detection algorithm (Redmon et al., 2016; Redmon and Farhadi, 2017, 2018; Bochkovskiy et al., 2020; Wang et al., 2023). YOLOv8 is the structure of YOLO family introduced in 2023, which performs target detection by means of a unique two-path prediction and tightly connected convolutional network. The algorithm employs a lightweight network structure while maintaining a high performance and is therefore efficient. In YOLOv8, the target detection task is decomposed into two independent subtasks, namely classification and localization. Each subtask has its own network path, which enables the algorithm to better handle targets of different sizes. In addition, YOLOv8 adopts the ideas of cascading and pyramiding, which enables the algorithm to deal with targets of different sizes. The feature extraction capability of YOLOv8 has been significantly improved compared to the previous model while maintaining a lightweight network structure. The main structure of YOLOv8 consists of Backbone, Neck, and Head, as shown in the **Figure 6**.

The challenge of evaluating and detecting fingered citron slices is constrained by the availability of computational resources, necessitating models that are structurally straightforward, exhibit minimal delay, and offer substantial data processing capabilities. Traditional lightweight networks, such as MobileNet (Howard et al., 2017), ShuffleNet (Zhang et al., 2018), and GhostNet (Han et al., 2020), leverage deep convolution or swarm convolution to distill spatial features from visual data. While the primary objective of these lightweight models is to diminish the count of floating-point operations (FLOP), there is a dearth of research that has explored models with a low rate of floating-point operations per second (FLOPS). It’s important to note that merely reducing the model’s parameters does not completely equate to a proportional boost in computational velocity. Consequently, some studies have attempted to engineer nimble and rapid neural network components through the use of deep convolution or group convolution. However, these attempts do not always accelerate model performance and can sometimes even increase latency. In light of these considerations, this paper proposes the substitution of the Darknet-53 backbone network in YOLOv8n with the FasterNet-T0 network. This change is intended to augment the model’s parameter count and computational capacity, thereby enhancing its computational efficiency.

In the YOLOv8 framework, the PAN-FPN structure is utilized, which represents an enhanced iteration of the classic FPN. This PAN-FPN configuration is designed to mitigate the issue of partial loss of localization details by integrating a bottom-up PAN architecture atop the FPN, thereby replenishing the missing localization data. While this approach does bolster the semantic and spatial information to an extent, the PAN-FPN structure is still susceptible to refinements. To more effectively tackle the limitations of PAN-FPN, particularly its challenges with handling large-scale feature maps and the loss of some pristine information in feature maps post-sampling, this study proposes a restructured feature fusion mechanism for YOLOv8n that draws inspiration from the BiFPN.

**Figure 7:**
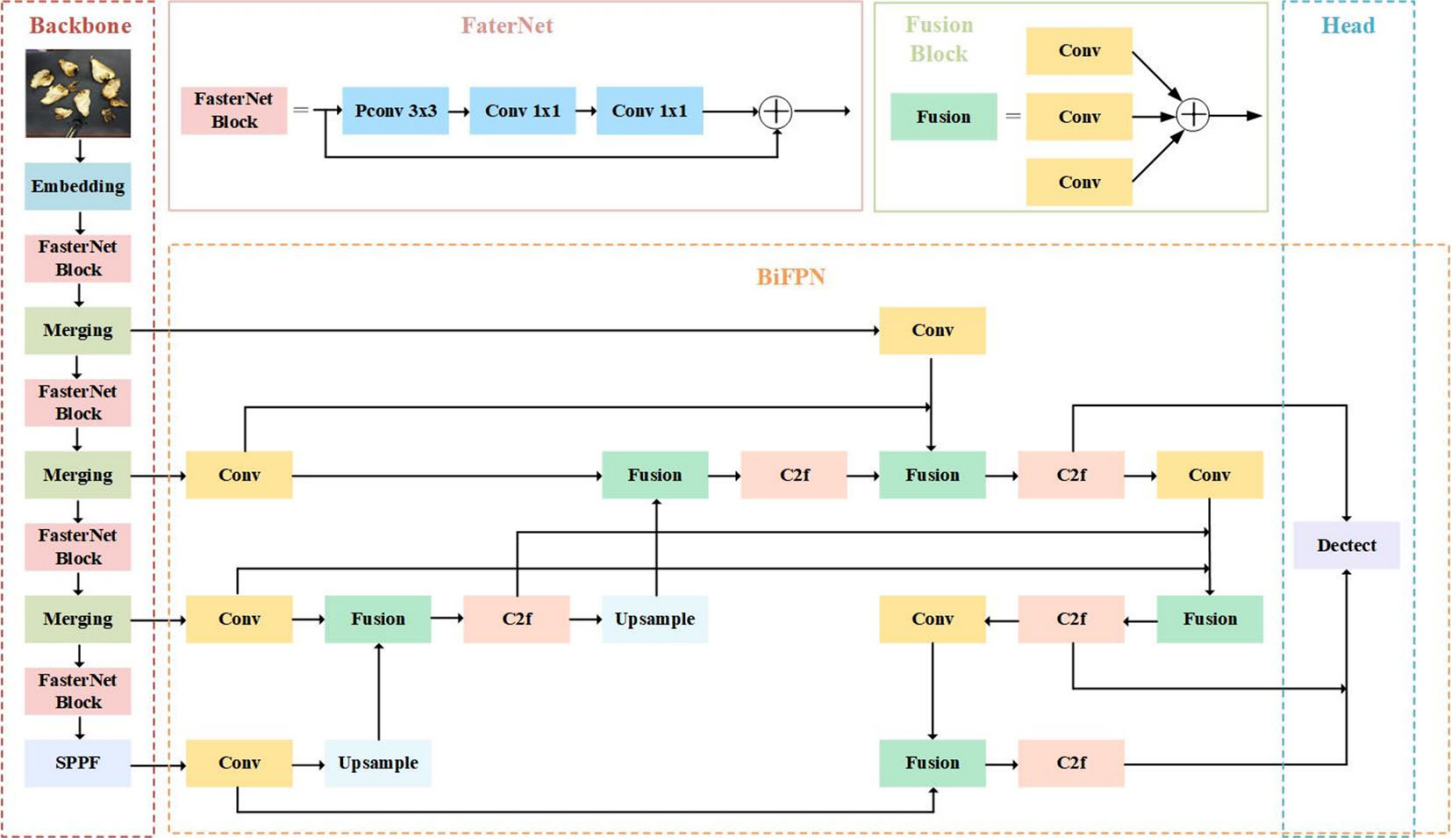
The main structure of YOLOv8-FCS.

The modified network structure is shown in **Figure 7**, which consists of the Backbone module with the improved FasterNet structure, the Neck module reconfigured according to the BiFPN structure, and the head module of the original model, which named YOLOv8-FCS.

YOLOv8-FCS is able to extract features better and obtain better recognition accuracy in comparison with the base YOLOv8n model, as verified in experiments in Section 3. The improved model effectively improves the overall detection performance of the model yet becomes complicated to a small extent. From the subsequent experiments, it can be seen that the model achieves better results in the task of grading and detecting fingered citron slices.

### 2.3 Experimental platform training parameters

In this study, the experiments were run on Windows 10. The framework image source used is PyTorch 1.8.1, with Python 3.8 as the training environment and the Compute Unified Device Architecture (CUDA) 11.1 as the computing architecture. The GPU for the hardware part is RTX 2080×1 with 8 GB of its video memory. the CPU is a 4-core, 8-thread 12th Gen Intel(R) Core(TM) i3–12100F 3.30 GHz with 16 GB of RAM.

The settings of each hyperparameter in this experiment are shown in **Table 2**. In order to balance the different shooting devices, the input shape value of the input model was standardized to 640×640, and all of them were trained for 200 epochs, and the mosaic enhancement was turned off at the last 10 epochs. The optimizer was SGD, the batch size was set to 8. The maximum learning rate was 1e-2, the minimum learning rate was 1e-4. The momentum is set to 0.937, and the IoU threshold for the mean Average Precision (mAP) is set to 0.7.

**Table 2:** Hyperparameter settings.

Parameter	Setting
Input Shape	(640, 640, 3)
Train Epoch	200
Close Mosaic	10
Batch Size	8
Workers	8
Optimizer	SGD
Maximum Learning Rate	1e-2
Minimum Learning Rate	1e-4
Momentum	0.937
IoU	0.7

## 3 Results

### 3.1 Evaluation indicators

To more accurately assess the precision and assurance levels of the model’s classifications, this study has chosen to employ the loss function curve (Loss), mean Average Precision (mAP), recall rate, the Giga Floating Point Operations Per second (GFLOPs), and the Frames Per Second (FPS) as the key metrics for evaluating the algorithm’s performance. The calculation of related evaluation indexes is shown in Equations 1–5.


(1) $$mAP=\frac{\sum_{1}^{N}AP}{N}=\frac{\sum_{1}^{N}\int_{0}^{1}P\left(R\right)dR}{N}$$

(2) $$Precision=\frac{TP}{TP+FP}$$

(3) $$Recall=\frac{TP}{TP+FN}$$

(4) $$Mean\;AveragePrecesion=\frac{\sum AveragePrecesion}{n\left(Class\right)}$$

(5) $$F-score=\frac{2\times Precision\times Recall}{Precision+Recall}$$

In our experiment, the True Positives (TP) denotes the count of instances that were accurately identified as positive instances, the True Negatives (TN) signifies the count of instances that were correctly classified as negative instances, the False Positives (FP) represents the instances that were incorrectly categorized as positive when they were actually negative, and the False Negatives (FN) corresponds to the instances that were mistakenly labeled as negative when they were positive. The Average Precision (AP) is calculated as the area beneath the Precision-Recall curve (P-R curve), which is a measure of the precision across various recall levels and represents the average precision. The mean Average Precision (mAP) is the mean value of the AP across different object categories. The *n* represents the total number of sample categories that were subjected to testing. The F-score is a statistical measure that balances the impact of precision and recall, providing a single score that encompasses both metrics for a more comprehensive assessment of the model’s performance.

### 3.2 Impact of data enhancement

**Figure 8:**
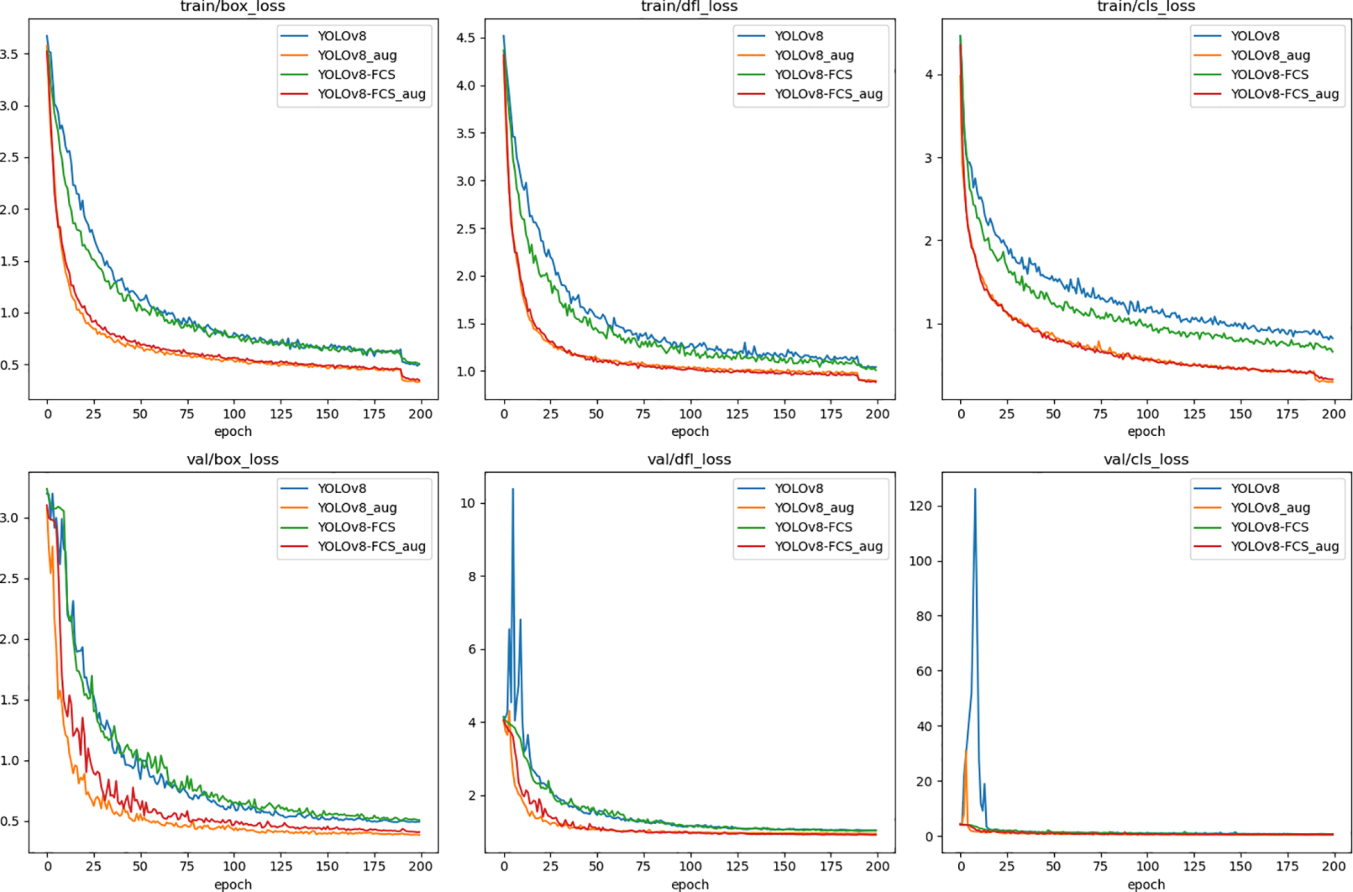
Loss chart before and after data augmentation.

To explore the impact of data augmentation on the outcomes of the experiments, this study employs both a non-augmented dataset and an augmented dataset to perform tests on the YOLOv8n and YOLOv8-FCS models respectively. Both datasets are segmented in a 6:2:2 ratio for training, validation, and testing, and the training is carried out for 200 epochs. The findings from these experiments are presented in **Figure 8**.

Observations indicate that, when compared with the results obtained from the non-augmented dataset, both the YOLOv8n and YOLOv8-FCS models exhibit an improvement in their correlation metrics on the augmented dataset, with an enhancement range of 2% to 5%. It is worth mentioning that in around 190 epochs of this study, the model experienced a significant decrease in loss value. This is to prevent the training images generated by Mosaic data augmentation from deviating from the true distribution of natural images and introducing a large number of inaccurate annotations. Therefore, Mosaic data augmentation was turned off in the last 10 rounds of training, and the model began to learn real image data. This transformation significantly reduces label errors, leading to a rapid decrease in training loss values. These experimental outcomes confirm that the data augmentation techniques applied in this study play a significant role in bolstering the accuracy of the model’s detection capabilities.

### 3.3 Comparison with YOLOv8 before improvements

**Figure 9:**
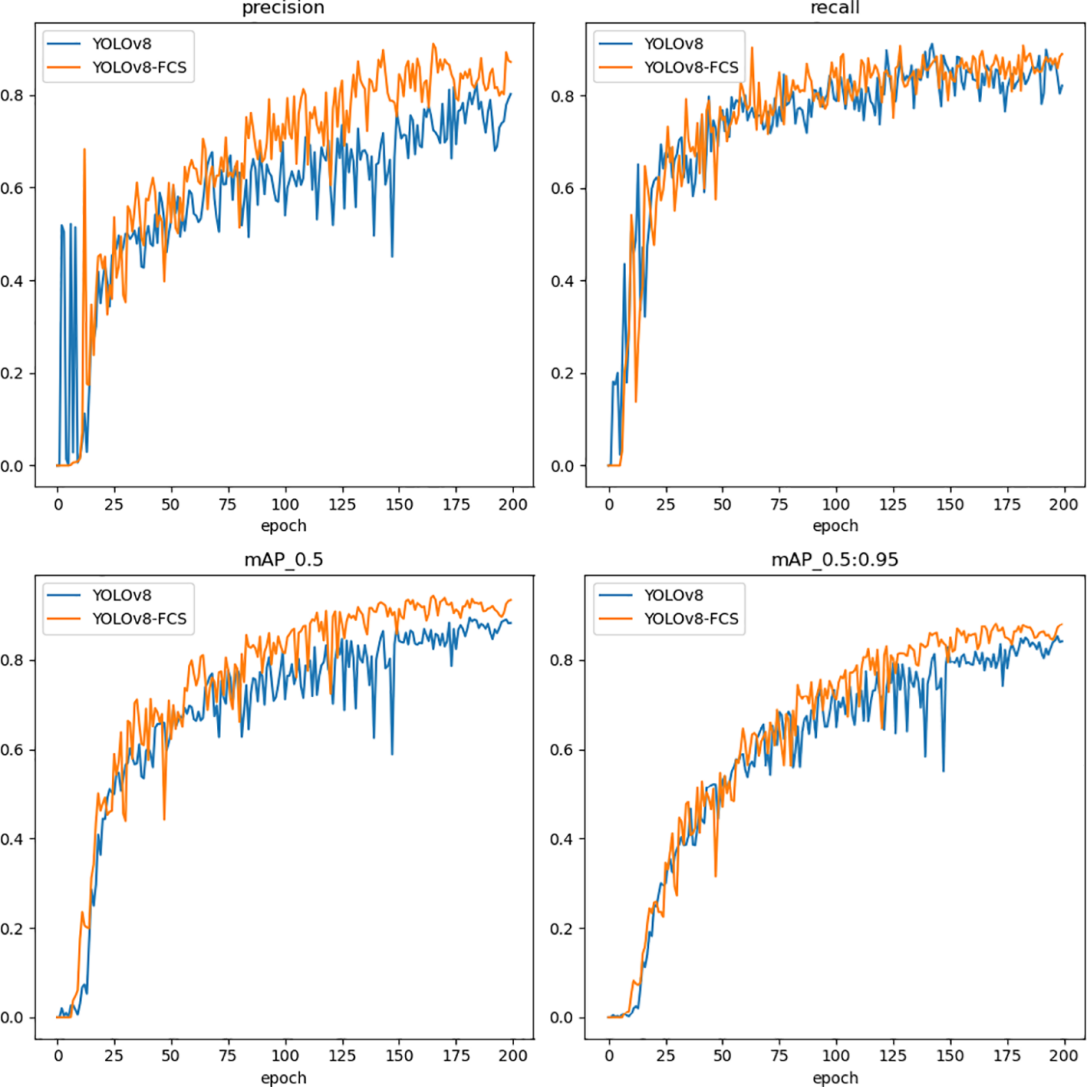
Comparison of the Evaluation indicator chart of YOLOv8 and YOLOv8-FCS.

**Figure 9** show the comparison of the main algorithmic performance evaluation metrics of the pre-improved YOLOv8n model and the improved YOLOv8-FCS. During the training process, the loss function of YOLOv8-FCS shows a faster convergence trend, as well as a lower training loss. In addition, the improved YOLOv8-FCS model gets better results in precision, recall, and mAP metrics. This indicates that the improvement of this experiment enables the model to learn the feature information related to the grading of fingered citron slices faster, so that the convergence speed of the model increases and the training loss decreases, which leads to better training effect and accuracy.

**Figure 10:**
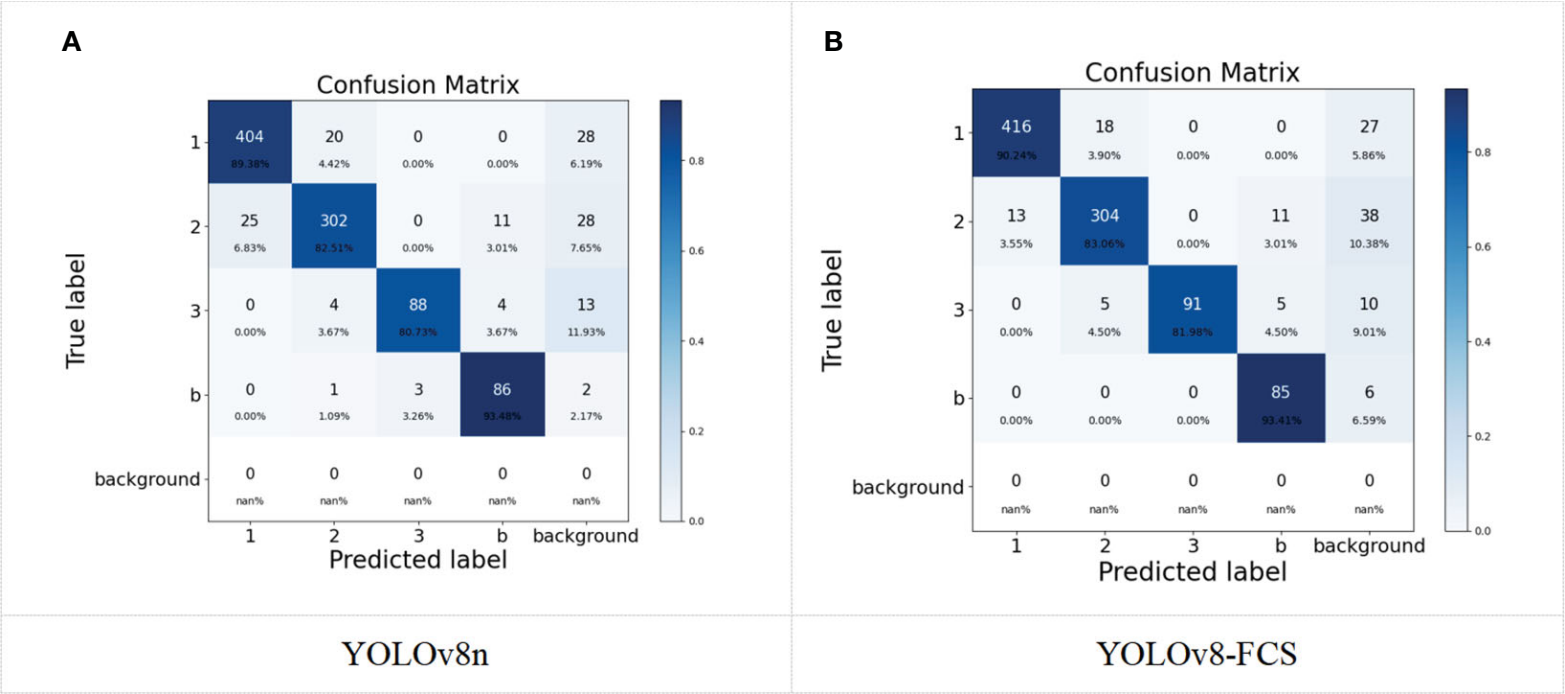
Confusion Matrix for YOLOv8n **(A)** and YOLOv8-FCS **(B)**.

**Figure 10** illustrates the confusion matrix for both the original and the enhanced models across four categories of fingered citron slices. In this matrix, each row corresponds to the true class of the samples, each column corresponds to the class predicted by the model, and the diagonal elements indicate the count of samples correctly classified for each class. The data reveals that there is a high rate of accurate predictions for the first and bad grades of fingered citron slices, whereas the second and third-grades have a comparatively lower accuracy in predictions. The confusion matrix highlights that there is a tendency for the model to confuse the first and second class goods. Upon examination of the images that led to incorrect classifications, it was observed that a significant number of these instances involved targets with occlusion or adhesion. Such conditions can result in certain features being obscured, causing defects and characteristics to be incorrectly identified due to the masking effect.

By comparing the confusion matrices of the models before and after the improvement, it can be found that the number of first-grade products misjudged as second-grade products is greatly reduced, which directly improves the judgment accuracy of first-grade products. At the same time, the number of second-grade and third-grade samples judged correctly are both improved to some extent. This is because we have redesigned the neck file of the model using the BiFPN structure to improve feature fusion capability. This suggests that the improvements in this experiment have a positive effect on the accurate identification of each class of fingered citron slices. However, from **Figure 10B**, it can be seen that the improved model has a slight decrease in the ability to distinguish between bad and third-grade products. This is because the weakened expression ability of the model in the feature extraction process for some subtle features of bergamot slices, resulting in some fuzzy recognition at the boundaries of these two categories. Overall, the prediction accuracy of the four categories of fingered citron slices is relatively high, indicating that the model is suitable for the classification detection task of fingered citron slices from a fixed perspective.

**Figure 11:**
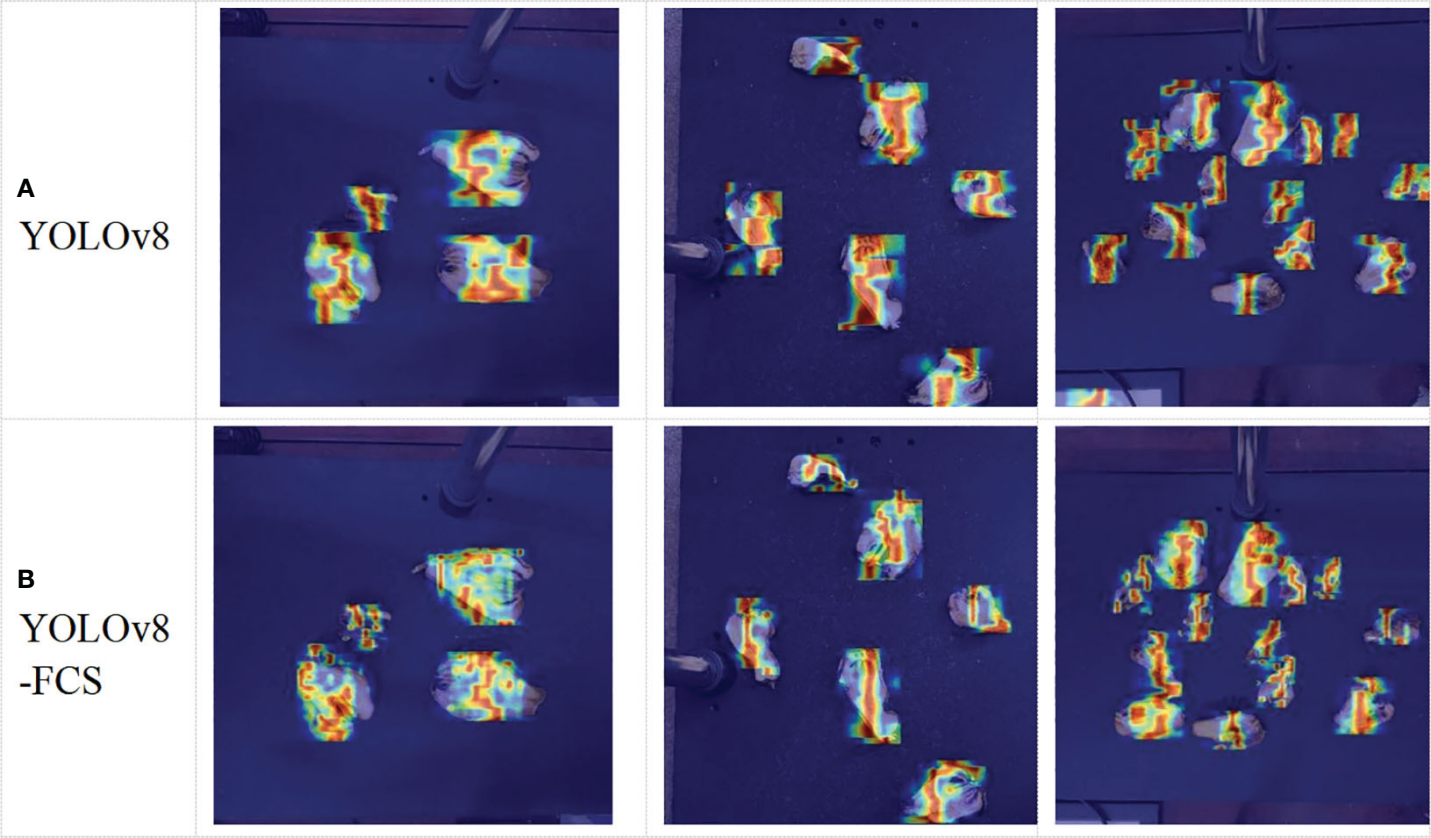
Heat maps of YOLOv8n **(A)** and YOLOv8-FCS **(B)**.

In order to further observe the effect of the improvement in this experiment, this study also tries to use HiRes-CAM (Esmaeili et al., 2021) to visualize the sample regions that are more concerned by the YOLOv8 and the improved YOLOv8-FCS model respectively, as shown in **Figure 11**. Different highlighted regions indicate the degree of influence of pixel points at different locations on the recognition results, where red highlighting indicates that the pixel values in the region have the greatest influence on the recognition results and get the highest convolutional attention. By comparison, we find that YOLOv8-FCS pays more attention to the target itself and focuses the attention constriction on the target. Furthermore, for the adhesion case, YOLOv8-FCS is able to distinguish the target better, instead of confusing the adhesion part. In addition, for problems such as misdetecting the background as the target, which existed in the YOLOv8n model, YOLOv8-FCS is able to avoid them to a large extent. In summary, it is further demonstrated that the YOLOv8-FCS model proposed in this paper can improve the learning ability of important features for grading samples of fingered citron slices.

In summary, the YOLOv8-FCS model proposed in this paper is superior to the YOLOv8n model in terms of comprehensive metrics, and is more suitable for the task of grading fingered citron slices, which is the focus of this experiment.

### 3.4 Ablation experiments

In order to validate the feasibility and effectiveness of the enhanced model, the improved methodology is divided into two key components, namely, replacing the FasterNet module in the backbone network of YOLOv8n and redesigning the PAN-FPN structure of YOLOv8n using the BiFPN structure. Four different ablation experiments were conducted in order to fully assess the impact of changes and combinations of individual modules on the performance of the algorithm. The results of these experiments are combined and summarized in **Table 3**.

**Table 3:** Results of ablation experiment.

Model	Precision	Recall	mAP	FPS	GFLOPs	ModelSize
YOLOv8n	0.939	0.909	0.967	**194.3**	8.1	5.95MB
YOLOv8-Fasternet	0.916	**0.961**	0.978	138.2	10.7	8.20MB
YOLOv8-BiFPN	0.931	0.943	0.973	169.1	**7.5**	**4.15MB**
YOLOv8-FCS	**0.961**	0.949	**0.981**	130.3	10.1	6.40MB

The bold values represent the most outstanding performance among the evaluation indicators.

The purpose of this experiment is to evaluate the effect of different components on the performance of the YOLOv8n model by observing the changes in precision, recall, computation volume (GFLOPs), model size, Frames Per Second (FPS) transmitted on the screen, and mean Average Precision (mAP). The original YOLOv8n model achieves 93.3% precision and 90.9% recall, with a mAP of 96.7% and FPS of 194.3, while maintaining a low computational effort (8.1 GFLOPs) and model volume (5.95 MB). This shows that YOLOv8n has been designed to be efficient and accurate.

The design goal of FasterNet is to improve the computational efficiency of the model. After introducing FasterNet as the backbone network (YOLOv8-FasterNet), the computational volume of the model increased to 10.7 GFLOPs, the size increased to 8.20 MB, the precision and recall were improved, the mAP increased to 97.8%, and the recall reached the highest value of 0.961 for this ablation experiment. Although FasterNet optimizes the computational efficiency, this optimization comes at the cost of increasing the size and complexity of the model. This is due to the fact that FasterNet introduces more parameters or more complex network structures, which increases the model’s representational power. The increased model complexity allows FasterNet to capture more features and details, which is directly reflected in improved precision and recall. Increased precision means that the model correctly identifies an increased percentage of targets, while increased recall means that the model is able to identify more real targets and reduces missed detections. The mAP is increased to show that the model performs well under a wide range of conditions. The recall rate reaches the highest value in the experiment, which further confirms the effectiveness of FasterNet in the target detection task.

BiFPN as a multi-scale feature fusion technique, is able to reduce the size and computational effort of the model while maintaining performance. When BiFPN is added as the neck structure (YOLOv8-BiFPN), the model volume reaches the lowest value of 4.15 MB for this ablation experiment, and the amount of computation is reduced to 7.5 GFLOP. This is due to BiFPN’s ability to efficiently integrate features at different scales, which reduces the need for a large number of parameters. Despite the reduction in model size and computation, the addition of BiFPN still results in a slightly better performance than the original model. This suggests that BiFPN does a good job of integrating multi-scale information and is able to improve the model’s ability to detect targets of different sizes. It is mentioned in the paper that the improvements in BiFPN are useful for a specific task that fingered citron slice grading detection. This is because the task requires accurate identification and classification of slices of different sizes and shapes, and BiFPN is able to provide just this kind of multi-scale feature fusion.

When the two improved components are used together (YOLOv8-FCS), the volume of the model is 6.40 MB and the computational volume increases to 10.1 GFLOPs, which is higher compared to the original YOLOv8n model but lower than the model that replaces the FasterNet structure alone. This suggests that the combination of the two improves computational efficiency while keeping the computational cost under control. Meanwhile, the improved precision and recall reached 96.1% and 94.9%, respectively. mAP was 98.1%, which was the highest among all models. This result shows that the combination of YOLOv8n, FasterNet and BiFPN maximizes the performance of the model and achieves a balance between high efficiency and high precision. Although the performance of the two improved models is lower than any of the improved as well as the base model in terms of FPS, it still reaches a respectable value of 130.3, which is fully adequate for deployment.

To summarize, the experimental results show that the detection performance of the YOLOv8n model can be significantly improved by reasonably increasing the number of parameters and computational volume of the model. In particular, the FasterNet module plays a key role in improving precision and recall, and BiFPN performs well in controlling the model volume and computation. Meanwhile, FasterNet and BiFPN optimize the feature extraction and multi-scale feature fusion capabilities of the model to different degrees. The combined use of these improved components provides an effective way to achieve more efficient and accurate target detection.

### 3.5 Comparison with other models

In order to fully evaluate the performance of the YOLOv8-FCS model, we compare it with several other popular target detection models, including RT-DETR (Zhao et al., 2023), YOLOv7-tiny (Wang et al., 2023), YOLOv5n, YOLOv5s, SSD (Liu et al., 2016) and FasterR-CNN (Ren et al., 2016). The experimental results of these models on the same dataset are shown in **Table 4**.

**Table 4:** Results of comparison test.

Model	Precision	Recall	mAP	FPS	ModelSize
SSD	0.836	0.899	0.907	26.6	92.1MB
FasterR-CNN	0.754	0.937	0.863	18.4	108MB
RT-DETR-resnet18	0.936	0.913	0.924	46.7	38.5MB
YOLOv5n	0.945	0.893	0.947	**190.2**	**5.02MB**
YOLOv5s	0.938	**0.957**	0.979	124.3	17.60MB
YOLOv7-tiny	0.955	0.948	0.977	143.8	12.30MB
YOLOv8-FCS	**0.961**	0.949	**0.981**	130.3	6.40MB

The bold values represent the most outstanding performance among the evaluation indicators.

As can be seen from **Table 4**, the mAP value of the YOLOv8-FCS model proposed in this paper reaches 98.1% with the same experimental setup. In contrast, RT-DETR-resnet18 (92.4%), the faster FasterR-CNN (86.3%), SSD (90.7%), YOLOv5n (94.7%), YOLOv5s (97.9%), and YOLOv7 (97.7%) are not as good as the YOLOv8-FCS model in terms of mAP. Comparison experiments show that the real-time detection rate (FPS) of both Faster R-CNN (18.4) and SSD (26.6) do not meet the real-time requirements. While YOLOv5n (190.2), YOLOv5s (124.3) and YOLOv7-tiny (143.8) perform well in this regard. The YOLOv8-FCS model (130.3) proposed in this paper does not have the highest real-time detection rate, but it fully meets the practical deployment requirements. YOLOv7-tiny and YOLOv5s are 1–2 of the size of this experimental model, but their detection accuracy, recall and mAP values are not gap with the YOLOv8-FCS model. In contrast, the size of the improved algorithm model proposed in this paper is 6.4 MB, which is much smaller than Faster R-CNN, RT-DETR-resnet18 and SSD models, and only slightly larger than the YOLOv5n model, but it performs much better in the evaluation metrics.

In summary, the YOLOv8-CFS model proposed in this paper not only meets the real-time detection requirements, but also improves the detection accuracy, minimizes the model size, and exchanges a very small arithmetic cost for a large performance improvement, which has higher generality and practical value. This result not only demonstrates the superiority of YOLOv8-CFS in target detection tasks, but also shows that the detection accuracy of the model can be significantly improved without significantly affecting the computational efficiency through our well-designed network structure improvement.

## 4 Discussion

### 4.1 Feasibility analysis

In order to promote the intelligent development of fingered citron slices industry, this study applied the YOLOv8n model to the task of grading and detecting fingered citron slices under the fixed perspective, and improved the YOLOv8n for the limitations, and finally obtained the YOLOv8-FCS fingered citron slices grading and detecting model. The results show that the YOLOv8-FCS model can accomplish the fingered citron slices grading detection task better.

The size of the YOLOv8-FCS network, as introduced in this study, is 6.4MB, marking an increment of merely 0.4MB over the standard YOLOv8 model. The model requires a mere 0.046 seconds to process a single image within the experimental setup, which includes a GPU (RTX 2080 with 8GB of video memory). The evaluation metrics of the refined model significantly surpass those of other prevalent models typically used for object detection tasks. The empirical findings indicate that our model aligns well with hardware deployment requirements, and its processing velocity satisfies practical demands. Consequently, the model demonstrated in this research holds a distinct accuracy advantage over existing models. It is capable of effectively executing the grading detection task for fingered citron slices from fixed viewpoints and holds promise for future integration into assembly lines or sorting machinery designed for fingered citron slices.

### 4.2 Contribution to the intellectualization of the fingered citron slices industry

At present, the majority of the fingered citron slices sector relies on conventional manual methods. These methods are characterized by low efficiency, substantial labor expenses, and a significant degree of subjectivity. Consequently, there is a pressing need to explore intelligent processing solutions within the fingered citron slices industry to address these shortcomings. Manual sorting of fingered citron slices is a labor-intensive task that requires a lot of manpower, and the use of mechanical grading helps to reduce the labor demand and can improve grading accuracy and avoid the influence of subjectivity. In this study, a deep learning algorithm is utilized to achieve the task of grading fingered citron slices detection under fixed viewpoint. We propose the YOLOv8-FCS model to complete the task of grading fingered citron slices, which has performed well in testing and can process 130 images per second (FPS=130.3). This processing speed not only meets the requirements of industrial applications, but also has the potential for practical deployment on fingered citron slices processing flowlines or automatic sorting machinery. The efficiency of the YOLOv8 FCS model means that it can significantly improve the automation level and production efficiency of the classification of fingered citron slices while maintaining high accuracy. In addition, the design flexibility and scalability of this model also provide possibilities for its application in different working environments, thereby providing strong technical support for the automatic grading of fingered citron slices and the automation of subsequent packaging, sales, and other processes.

Non-intrusive examination techniques have become extensively integrated into agricultural practices and are often merged with a variety of innovative technologies (Kondo, 2010), signaling a trend towards the automation of post-harvest activities as the next frontier in agricultural advancement. The utilization of machine vision technology enables swift and dependable evaluation of agricultural commodities like fruits and vegetables, with non-destructive approaches that can minimize financial losses while simultaneously boosting productivity and economic gain. In the context of this research, the multi-input model has been applied to the task of grading and detecting fingered citron slices, representing an instance of processed produce in sliced form. As such, the model exhibits considerable promise for extending its utility to the grading of other sliced herbs and potentially for the quality assessment of fruits and vegetables more broadly.

### 4.3 Limitations and future work

Compared with the original YOLOv8n model, the improved YOLOv8-FCS model in this paper improves the accuracy of fingered citron slices grading detection. Nevertheless, a small number of false detection exist, which may be attributed to the following factors. The first reason is related to the features of the images. The difference features between the grades of fingered citron slices are more variable, which increases the detection challenge. The second reason is the complexity of the datasets, where the captured images not only have scattered targets of fingered citron slices, but also partially adhered and overlapped. These factors tend to mask the differential features of fingered citron slices, leading to false detection. The third problem is the limitation of the datasets. The datasets of this experiment were all taken from the same batch of fingered citron slices, which may not be representative enough. The above problems indicate that the model has some room for improvement in dealing with the problem of datasets with complex features. In the future, we will evaluate the performance of the datasets under more equipment shots and try to add other batches and provenances of fingered citron slices to enhance the representativeness of the datasets.

## 5 Conclusion

In this study, we apply deep learning techniques to the grading detection of fingered citron slices, and improve the YOLOv8n model to get the YOLOv8-FCS model which is more effective in grading detection of fingered citron slices. Firstly, we enhanced and labeled the image data of fingered citron slices collected under the fixed viewing angle to form the fingered citron slices datasets. Then we replace the YOLOv8n backbone structure with the FasterNet main module to improve the computational efficiency in the feature extraction process. The PAN-FPN structure used in the original model is redesigned with the BiFPN structure in order to balance the computational volume and model size while making full use of the high-resolution features to expand the sensory field of the model. In the end, the detection accuracy is improved significantly at a small cost of arithmetic power. The main conclusions are as follows:

(1) The mAP value of the YOLOv8-FCS model proposed in this study reaches 98.1%, which is improved by 1.4% compared with the YOLOv8n model. It is found that the YOLOv8-FCS model is able to achieve higher detection accuracy with a very small model size and computation enhancement, which alleviates the YOLOv8n model’s misdetection to a certain extent. Therefore, we believe that the improved method we used is effective and the YOLOv8-FCS model can effectively provide technical support for grading fingered citron slices.
(2) Following a comparative evaluation, it has been observed that the YOLOv8-FCS model outperforms current mainstream object detection networks in terms of accuracy for the task of grading detection of fingered citron slices. The model size of 6.4M is acceptable. The arithmetic capacity is 10.1GLOPs, which is very low, and the FPS is 130, which can meet the practical use requirements. The experimental results show that YOLOv8-FCS can accurately accomplish the task of fingered citron slices grading detection and can run on lightweight devices.
(3) The method proposed in this study can well accomplish the grading detection of fingered citron slices under the fixed viewpoint, which effectively reduces the problems of low efficiency, high cost, and great influence by subjectivity that exist in the traditional manual detection. Boasting both high precision and real-time capabilities, this model is well-suited to fulfill the grading requirements of agricultural producers and small to medium-sized enterprises. It holds significant practical value for application within the industry dedicated to the grading of fingered citron slices, contributing to the advancement of a standardized and efficient fingered citron slices market.
(4) Given that the primary focus of this research is on the grading detection of fingered citron slices, a type of herbaceous slice, future work may explore the potential of adapting the methodologies presented in this paper to the evaluation of other sliced agricultural products. In addition, we will also consider the use of more informative sensors such as multispectral cameras for image acquisition in order to grade fingered citron slices more accurately, authoritatively, and efficiently by combining the scales of active ingredient content.

## Data availability statement

The raw data supporting the conclusions of this article will be made available by the authors, without undue reservation.

## Author contributions

LZ: Writing – original draft, Writing – review & editing, Conceptualization, Formal analysis, Investigation, Methodology, Software, Supervision, Visualization. PL: Data curation, Methodology, Supervision, Writing – original draft. SD: Investigation, Validation, Writing – review & editing. TL: Validation, Writing – review & editing. KQ: Methodology, Supervision, Writing – original draft. JM: Writing – original draft, Funding acquisition, Methodology, Project administration, Resources, Supervision.
